# Traffic Scene Semantic Segmentation Enhancement Based on Cylinder3D with Multi-Scale 3D Attention

**DOI:** 10.3390/s25216536

**Published:** 2025-10-23

**Authors:** Yun Bai, Xu Zhou, Yuxuan Gong, Yuanhao Huang

**Affiliations:** 1 School of Aviation, Inner Mongolia University of Technology, Hohhot 010051, China; 2 State Key Lab of Intelligent Transportation System, School of Transportation Science and Engineering, Beihang University, Beijing 100191, China

**Keywords:** three-dimensional point cloud, semantic segmentation, multi-scale feature extraction, deep learning

## Abstract

With the rapid development of 3D sensor technology, point cloud semantic segmentation has found widespread applications in autonomous driving, remote sensing, mapping, and industrial manufacturing. However, outdoor traffic scenes present significant challenges: point clouds are inherently disordered, unevenly distributed, and unstructured. As a result, traditional point cloud semantic segmentation methods often suffer from low accuracy and unstable performance in complex tasks such as semantic segmentation and object detection. To address these limitations, this paper proposes an improved point cloud semantic segmentation method based on Cylinder3D. The proposed approach integrates the PointMamba and MS3DAM modules, which enhance the model’s ability to capture global features while preserving local details, thereby improving adaptability and recognition across multiple feature scales. Furthermore, leveraging the linear computational complexity of Mamba enables the method to maintain high efficiency when processing large-scale point cloud data. In addition, incorporating the KAT module into the encoder improves the model’s perceptual capacity and robustness in handling point clouds. Experimental results on the SemanticKITTI dataset demonstrate that the proposed method achieves a mean Intersection over Union (mIoU) of 64.98%, representing a 2.81% improvement over Cylinder3D, thereby confirming its superior segmentation accuracy compared with existing models.

## 1. Introduction

With the advancement of sensor technology, the performance of LiDAR, a key sensor for acquiring point cloud data, has continuously improved [1,2]. Three-dimensional LiDAR sensors have become indispensable components of autonomous driving systems [3], and the progress in 3D sensing has facilitated the widespread use of point clouds in diverse fields, including autonomous driving, mapping, and industrial manufacturing [4]. Point cloud data represent spatial geometry and object surfaces through collections of 3D points [5]. Compared with traditional 2D image data, point clouds offer richer information and a more comprehensive representation of complex scenes [6]. However, their inherent disorder, uneven distribution, and unstructured characteristics pose significant challenges for traditional convolutional neural networks, which struggle to efficiently and accurately perform segmentation on 3D point clouds. In traffic environments, these challenges result in reduced accuracy and unstable segmentation performance. To address this, recent studies have proposed various deep learning frameworks for 3D point cloud segmentation [7], aiming to enhance segmentation accuracy by exploiting the unique characteristics of point cloud data. In traffic scenarios, the design of accurate and efficient algorithms, along with optimized network architectures, can improve the speed and precision of segmenting moving vehicles, pedestrians, and other dynamic objects, thereby contributing to traffic safety on railways and highways.

Current approaches to 3D point cloud processing can be broadly categorized into three types [8]: projection-based, voxel-based, and point-based methods. Projection-based methods map 3D scenes onto 2D planes, thereby transforming 3D problems into 2D ones and enabling the use of well-established 2D convolutional neural networks, such as SqueezeNet [9]. However, this projection inevitably results in information loss. Voxel-based [10] methods partition 3D point cloud data into fixed voxels, creating a structured representation that allows the application of 3D convolutional neural networks. Despite this advantage, voxelization often leads to information loss and high computational complexity. In contrast, point-based methods operate directly on raw point cloud data, preserving fine-grained structural details and minimizing information loss.

In recent years, Transformer-based architectures have emerged as a prominent research focus for point cloud understanding. Guo et al. [11] proposed a Transformer-based approach incorporating an attention mechanism, which, while effective for global modeling, substantially increases computational complexity and model parameters. Stratified Transformer further enhances the modeling of local geometric details and long-range contextual dependencies through a hierarchical attention mechanism. Meanwhile, state-space sequence models (SSMs), such as S4 and its recent variant Mamba, have demonstrated the capacity to model long sequences with linear complexity. These approaches address the quadratic complexity limitation of standard Transformers and offer potential efficiency advantages for high-resolution point clouds.

Despite these advances, the direct application of existing Transformer or SSM frameworks to outdoor traffic scenes remains challenging. LiDAR data in traffic environments are more complex than indoor scans or object-level datasets: point cloud distributions are highly inhomogeneous, object scales vary significantly, and scenes contain both dynamic and static targets. Moreover, autonomous driving imposes strict latency and memory constraints, necessitating segmentation models that are both accurate and real-time capable.

Cylinder3D effectively addresses the challenge of uneven point distribution in outdoor LiDAR scenes through a cylindrical partitioning strategy, enabling better geometric representation of objects. However, it still struggles to capture multi-scale contextual relationships and fine-grained local details, leading to unstable segmentation results in complex traffic scenes.

To overcome these limitations, this study proposes CMPNet, which builds upon the Cylinder3D [12] framework and proposes a novel Multi-Scale 3D Attention Module (MS3DAM) to enhance both local feature extraction and global context modeling. By integrating features across multiple spatial scales, CMPNet achieves more accurate segmentation of objects with varying sizes, thus improving the robustness and accuracy of semantic segmentation in real-world traffic environments.

## 2. Related Work

### 2.1. Traditional Point Cloud Semantic Segmentation Methods

#### 2.1.1. Point-Based Methods

The point-based approach uses raw point cloud data as input to deep learning models. While this method preserves data integrity, it is insufficient for learning local features and cannot effectively address the uneven point cloud density in complex scenes. To address this limitation, Qi et al. proposed PointNet++ [13], an extension of PointNet [14] that introduces a hierarchical feature learning framework to capture local point cloud information. PointNet++ aggregates local features through farthest point sampling and progressively constructs global feature representations, demonstrating strong generalization across varying densities and resolutions. However, for large-scale outdoor point cloud data, its multi-scale grouping and recursive aggregation operations result in high computational complexity and memory consumption. To address these issues, RangeNet++ [15] projects 3D point clouds onto 2D images via spherical projection, segments the resulting depth maps using 2D convolutional neural networks, and maps the results back to 3D space using a KNN-based post-processing algorithm. This approach enables fast and accurate semantic segmentation suitable for autonomous driving applications. Nevertheless, semantic segmentation of small targets remains imprecise, and differentiating similar parts of distinct objects remains challenging. For large-scale point clouds, RandLA-Net [16] significantly reduces computational complexity and memory usage by introducing random local regions and a local feature aggregation mechanism. Additionally, it enhances the understanding of complex geometric structures through multi-scale feature fusion and extracts global contextual information using a global feature extraction module, which is then integrated with local features to improve overall segmentation performance.

Point-based methods effectively preserve the structure of the original point cloud. However, they often emphasize either global or local feature extraction while neglecting the correlations among points, which leads to lower accuracy when segmenting adjacent objects in a scene.

#### 2.1.2. Voxel-Based Methods

Voxel-based methods for point cloud semantic segmentation convert point cloud data into structured 3D grids and leverage established 3D convolutional neural networks for feature extraction, enabling efficient semantic segmentation. By structuring the inherently irregular and sparse point cloud data, these methods allow traditional 2D and 3D convolutional networks to be directly applied to point cloud processing tasks. For large-scale point cloud data, the SPVCNN [17] model introduces 3D-NAS, demonstrating significant improvements in mean Intersection over Union (mIoU). However, voxelization methods exhibit limited effectiveness for outdoor point clouds. To address this limitation, Cylinder3D [18] replaces traditional voxelization with cylindrical partitioning, which better accommodates the sparsity and varying density of large-scale point clouds. Cylinder3D also incorporates two asymmetric residual blocks to preserve features of cuboid-shaped objects while reducing computational cost and memory usage. MinkUNet [19] introduces a 4D spatio-temporal convolutional neural network for processing 3D videos, which in some cases outperforms conventional 3D CNNs in speed. Furthermore, it proposes a generalized sparse convolution algorithm for high-dimensional point cloud data, effectively enhancing computational efficiency and overall model performance through the use of sparse tensors. MSF-CSCNet [20] builds upon Cylinder3D by incorporating multi-scale voxel feature fusion and channel context modeling, thereby substantially enhancing both the accuracy and robustness of point cloud semantic segmentation. However, this approach exhibits a higher dependency on computational resources, and its adaptability across diverse scenes remains constrained.

### 2.2. Channel Attention and Spatial Attention Features

Different channels in a feature map correspond to different objects. Channel attention [21] explicitly models the dependencies between channels, adaptively determines the importance of each channel, and assigns different weights to enhance important features while suppressing less relevant features. Channel attention was first introduced by SENet [22], which aggregates global spatial information through two operations, Squeeze and Excitation, to learn channel importance, significantly improving image classification performance. Building on this, CBAM [23] enhances convolutional neural network feature representations by incorporating both channel and spatial attention mechanisms. Coordinate Attention [24] further combines positional information with channel attention through a coordinate attention module, enabling the model to more accurately locate and recognize objects of interest while maintaining low computational overhead.

The attention mechanism [25] aims to enhance a model’s representational capacity and overall performance by learning the relative importance of different parts of the input data and dynamically adjusting its focus accordingly. Spatial attention [26], in particular, captures relationships among different spatial locations within the input, and has been widely applied in domains such as image analysis, natural language processing, and video understanding. It improves the model’s ability to interpret and represent complex data. However, most existing designs apply self-attention directly to 2D feature maps, generating attention matrices based on isolated query–key pairs at each spatial location, without fully leveraging the rich contextual information surrounding the key. To address this limitation, the Contextual Transformer module (CoT) [27] incorporates contextual information into the key representation to learn a dynamic attention matrix, thereby enhancing the model’s representational power effectively. DANet [28] incorporates a spatial attention mechanism to model the contextual information surrounding each pixel, thereby enhancing its ability to capture the shape and edge characteristics of objects. By effectively modeling rich contextual dependencies within local features, the method leads to substantial improvements in segmentation performance.

### 2.3. Multi-Scale Dilated Convolution and Receptive Field

Dilated convolution [29] is primarily used to enlarge the receptive field, enabling segmentation tasks to capture broader context without increasing the number of parameters [30]. However, in practice, it often suffers from the grid effect, which leads to the loss of local information and limits the relevance of features captured from distant regions. To address these issues, this study adopts multi-scale dilated convolution, where each convolution branch employs a distinct dilation rate. This strategy enables the model to effectively capture information at multiple scales, thereby broadening the receptive field and improving the extraction of spatial information across diverse ranges. By setting varied dilation rates, the model is able to enhance its perception of spatial structures at different levels, ensuring both comprehensive feature representation and stronger contextual awareness.

By employing different expansion rates, the model can capture spatial structures at multiple scales, enabling both global feature representation and enhanced contextual modeling. In practice, smaller expansion rates emphasize local geometric details, whereas larger rates facilitate the capture of long-range dependencies in sparse traffic scenarios; medium rates provide a balance between these extremes. Although inflated convolutions can expand the receptive field without increasing the number of parameters, excessively large expansion rates may compromise local accuracy and reduce computational efficiency due to sparse sampling. Such trade-offs have been investigated in previous semantic segmentation studies, including DeepLab’s multiscale approach for 2D images, and have proven effective in 3D point cloud segmentation. To achieve a reasonable balance between accuracy and efficiency, we evaluated several combinations of expansion rates and determined that the combination of 3, 12, and 39 yields optimal multiscale feature modeling. This empirical selection was further validated through ablation experiments, where alternative configurations consistently resulted in reduced segmentation performance.

Although extensive research has been conducted on point cloud semantic segmentation, many algorithms still rely on a single channel output and lack effective information exchange between channel groups, which limits the expressive power of neural networks. To address this limitation, this paper integrates channel attention and spatial attention mechanisms into Cylinder3D, enhancing feature representation in key regions and improving overall model accuracy.

## 3. Methodology

### 3.1. CMPNet

**Figure 1:**
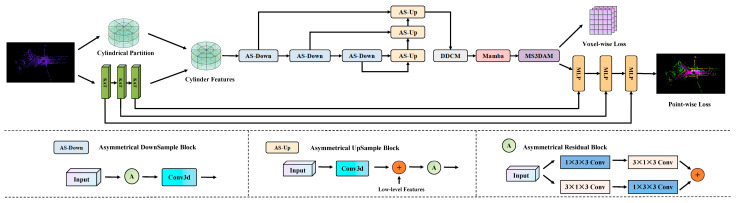
The CMPNet network structure.

Cylinder3D serves as the baseline architecture for this study. It projects irregular LiDAR point clouds into a cylindrical coordinate system to mitigate the uneven point density problem and employs voxel-based feature encoding and 3D sparse convolution to perform semantic segmentation. However, the original Cylinder3D framework faces three main limitations: (1) its voxel feature encoder, built upon standard MLPs, struggles to capture complex local spatial relationships; (2) it lacks an effective mechanism for multi-scale feature extraction and long-range dependency modeling; and (3) its normalization scheme exhibits limited robustness when handling sparse or highly unstructured outdoor scenes. To address these issues, we propose an improved point cloud semantic segmentation framework named CMPNet, which systematically enhances the feature representation capability and robustness of the Cylinder3D architecture. As shown in Figure 1, CMPNet retains the cylindrical partitioning strategy of Cylinder3D while integrating three functional modules: PointMamba [31], KAT [32] and MS3DAM.

Specifically, the input raw point cloud is first processed by the KAT encoder, which replaces the conventional Voxel Feature Encoder (VFE) in Cylinder3D. The KAT module introduces the GR-KAN mechanism to replace standard multilayer perceptrons (MLPs) with group rational functions, allowing it to capture intricate local spatial dependencies that conventional VFEs fail to model effectively. GR-KAN employs rational functions instead of B-splines, making it more compatible with GPU architectures and improving computational efficiency. In addition, GR-KAN reduces the number of model parameters and computational cost through parameter sharing, while its variance-preserving initialization enhances training stability. Within the KAT module, layer normalization is applied to stabilize the input distribution and maintain consistent feature scaling, thereby preserving the spatial structure of point cloud features during training.

After feature encoding, the processed features are redistributed through cylindrical partitioning to achieve a balanced spatial distribution. Next, an asymmetric 3D sparse convolutional network is employed to extract local geometric structures. Within this network, we introduce the MS3DAM, a newly designed component that applies multi-scale dilated convolutions and attention fusion to capture both fine-grained local details and global contextual cues, compensating for the limited multi-scale modeling capability of the original Cylinder3D.

Finally, to enhance global context perception and computational efficiency, the PointMamba module is incorporated. Its sequence-based feature aggregation enables long-range dependency modeling with linear complexity, significantly reducing the computational cost in large-scale outdoor traffic scenes.

Through these targeted enhancements, CMPNet effectively addresses the architectural limitations of Cylinder3D, achieving superior segmentation accuracy, robustness, and efficiency.

### 3.2. MS3DAM

In point cloud semantic segmentation tasks, objects often appear in various sizes and shapes. Conventional convolutional layers employ a fixed receptive field, which substantially restricts their ability to capture multi-scale features. To address this limitation, we design the MS3DAM that applies multiple dilation rates in parallel and integrates channel and spatial attention mechanisms to enhance feature representation [33].

First, MS3DAM enlarges the receptive field. Traditional convolutional networks expand the receptive field by stacking multiple convolutional layers, which significantly increases computational cost and may dilute important information. In contrast, dilated convolution, particularly with varying dilation rates, effectively enlarges the receptive field without reducing resolution, thereby enabling the model to capture broader contextual information.

Second, MS3DAM enhances the capture of multi-scale information. In tasks such as semantic segmentation and object detection, where objects differ greatly in size and shape, parallelizing convolutions with multiple dilation rates allows the network to simultaneously extract features at different scales. This improves the model’s adaptability and recognition of features across diverse spatial ranges.

Global average pooling [34] effectively captures the global features of an entire image, which is crucial for understanding the overall scene structure. Integrating this global information with local features enables the network to better interpret the relationship between individual image regions and the whole, thereby facilitating more accurate predictions.

In convolutional neural networks, different channels typically represent distinct features or patterns. However, when processing complex images, some channels contain more useful information than others, and conventional convolutional operations cannot dynamically adjust their importance. Similarly, spatial locations vary in significance; for instance, regions containing target objects are more critical than background areas. Traditional convolutions, however, treat all spatial locations equally and fail to emphasize key regions. To address these limitations, channel attention is introduced to evaluate and adjust the importance of each channel, thereby strengthening informative feature channels and suppressing less relevant ones. In addition, spatial attention is applied to highlight crucial regions within the image, enabling the model to focus on local areas that exert the greatest influence on the task outcome.

Finally, to enhance overall model performance, multi-scale features must be effectively integrated with the attention mechanism, as using either technique alone is insufficient. By concatenating the output features of multi-scale dilated convolutions along the channel dimension and applying both channel and spatial attention mechanisms for weighting, the module fuses features from multiple perspectives. Combining different dilated convolution layers with global features ensures that the network captures both local details and global context. This design produces more comprehensive network outputs and improves the model’s generalization across diverse scenarios. Additionally, feature dimensionality reduction via the final 1 × 1 × 1 convolution reduces computational cost and integrates information from different branches, generating a more effective feature representation for the downstream task.

**Figure 2:**
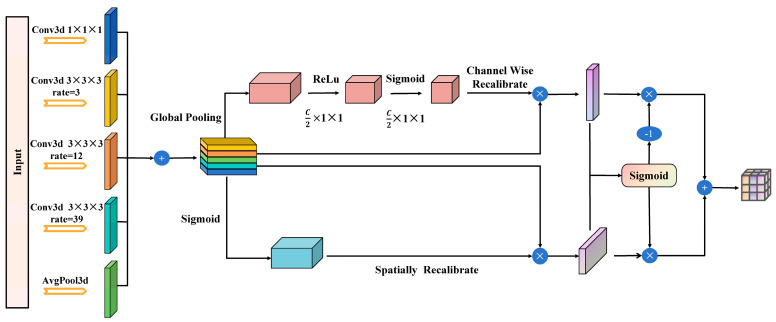
MS3DAM frame diagram.

The MS3DAM module, as illustrated in Figure 2, is designed to enhance feature representation by integrating multiple dilation rates and combining channel and spatial attention mechanisms. This architectural design effectively addresses the requirement to capture both fine-grained local information and broad contextual information within an image, which is essential for tackling complex visual tasks, including semantic segmentation in outdoor traffic scenarios and object detection applications.

This module represents an integrated combination of multi-scale dilated convolutions with channel and spatial attention mechanisms. It performs feature extraction across multiple scales through five parallel convolutional branches, each configured with distinct dilation rates to expand the receptive field and capture spatial information at varying ranges. The first branch employs a 1 × 1 × 1 convolutional kernel to directly extract features without altering spatial resolution. The second branch uses a 3 × 3 × 3 convolutional kernel with a dilation rate of 3, moderately enlarging the receptive field. The third branch applies a 3 × 3 × 3 convolutional kernel with a dilation rate of 12, further expanding the receptive field to capture a broader range of contextual information. The fourth branch achieves the widest receptive field using a 3 × 3 × 3 convolutional kernel with a dilation rate of 39. Finally, the fifth branch incorporates a global average pooling operation to extract global contextual features, thereby enhancing the model’s comprehension of the overall scene layout.

Subsequently, the multi-scale features extracted from the five distinct branches are concatenated along the channel dimension to form a unified feature map [35]. This combined feature map is then simultaneously refined using both the channel attention mechanism and the spatial attention mechanism. Within the channel attention module, global average pooling is applied to the synthesized feature map to obtain channel-wise global features. The importance weights for each channel are then learned through two fully connected layers employing ReLU and Sigmoid activation functions, respectively. These weights are finally multiplied by the original feature map in a channel-wise manner to implement channel weighting. In the spatial attention module, the combined feature map is globally pooled along the channel dimension to generate a spatial feature map. The importance weights for each spatial location are subsequently learned using a 1 × 1 × 1 convolution followed by a Sigmoid activation function. These weights are applied to the original feature map in an element-wise manner to achieve spatial weighting.

Finally, the output feature maps obtained from the channel attention and spatial attention mechanisms are fused through an element-wise addition operation to produce the enhanced feature maps. At this stage, the feature maps calibrated by the channel and spatial attention mechanisms are each combined with the original merged feature map via element-wise addition to integrate and reinforce the relevant features. The resulting enhanced feature maps are subsequently passed through a 1 × 1 × 1 convolutional layer for dimensionality reduction and feature integration, generating the final output feature representations.

By combining multi-scale dilated convolutions with channel and spatial attention, MS3DAM effectively captures both local details and global contextual information. This design addresses the limitations of Cylinder3D in multi-scale feature extraction and global perception, providing more comprehensive and robust features for semantic segmentation in complex outdoor traffic scenarios.

### 3.3. PointMamba

**Figure 3:**
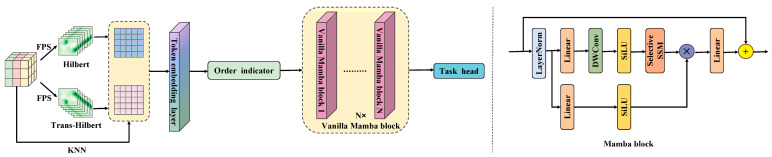
PointMamba frame diagram.

Cylinder3D employs cylindrical partitioning and 3D sparse convolutions to extract local geometric features from point clouds. However, it lacks an efficient mechanism for modeling long-range dependencies across large-scale point cloud sequences, which limits its capability to capture global contextual information in complex outdoor scenarios. To address this limitation, we introduce PointMamba, which offers linear computational complexity while maintaining the ability to model long sequences, as illustrated in Figure 3. Mamba [36] is based on a state space model [37] (SSM) that incorporates a selectivity mechanism, enabling the model to capture contextual information relevant to the inputs. The formulation of SSM is defined as follows:

(1) $$h\prime \left(t\right)=\bar{A}h\left(t\right)+\bar{B}x\left(t\right)$$

(2) $$y\left(t\right)=Ch\left(t\right)$$

where $\bar{A}\in R^{N\times N}$ denotes the state matrix, and $\bar{B}\in R^{N\times 1}$ and $C\in R^{1\times N}$ represent the mapping parameters. Both $\bar{A}\in R^{N\times N}$ and $\bar{B}\in R^{N\times 1}$ are determined using the zero-order hold (ZOH) rule:

(3) $$\bar{A}\in R^{N\times N}=exp\left(A\Delta \right),$$

(4) $$\bar{B}\in R^{N\times 1}={\left(A\Delta \right)}^{-1}\left(exp\left(A\Delta \right)-E\right)\cdot \Delta B,$$

Building on SSM, the SelectiveSSM (S6) framework makes the parameters $\bar{B}$, C and Δ functions of the inputs, effectively converting the SSM into a time-varying model and allowing the network to selectively focus on or filter out information. However, due to the inherent irregularity and sparsity of point clouds, Mamba’s unidirectional modeling encounters difficulties when processing point cloud data. These challenges can be effectively mitigated through the reordering strategy implemented in PointMamba.

When processing 3D point cloud data, PointMamba employs two types of space-filling curves, Hilbert [38] and Trans-Hilbert, to scan the key points, thereby preserving spatial localization effectively. Based on this, K-nearest neighbors (KNN) is applied to generate point patches, which are subsequently fed into the token embedding layer to produce serialized point tokens. To efficiently handle the inherently disordered structure of point clouds, a reordering strategy is applied to the point tokens, providing a more geometrically coherent scanning order. The reordered tokens are then input into an encoder composed of stacked Mamba modules [39], where feature extraction is performed via selective state-space modeling. This approach enhances the model’s global modeling capability and significantly reduces computational resource consumption, while retaining linear computational complexity.

Within CMPNet, PointMamba works synergistically with the KAT encoder and the MS3DAM module to complement local geometric feature extraction with global sequence modeling, forming an integrated and improved point cloud semantic segmentation framework based on Cylinder3D.

### 3.4. KAT

**Figure 4:**
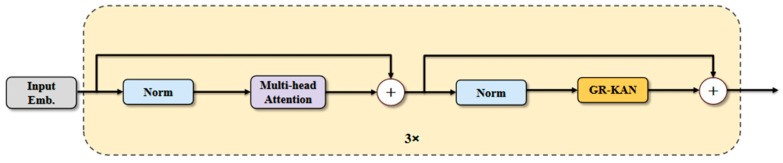
KAT frame diagram.

Traditional Voxel Feature Encoders (VFE) employ standard multilayer perceptron (MLP) in point cloud segmentation tasks, but they often struggle to capture the intricate spatial relationships inherent within point clouds, thereby limiting the model’s feature representation capability. Furthermore, conventional normalization methods exhibit limited robustness when applied to sparse point clouds. To address these limitations, we introduce the KAT module and replace the traditional MLP with the GR-KAN [40] module, as illustrated in Figure 4. GR-KAN utilizes rational functions in place of the B-splines [41] employed in the conventional KAN [42], which more effectively aligns with GPU architectures and enhances both computational efficiency and compatibility. In addition, GR-KAN [43] substantially reduces the number of model parameters and computational requirements through parameter sharing. Concurrently, variance-preserving initialization within GR-KAN further improves the stability of model training. The formula of GR-KAN is defined as follows:

(5) $$GR-KAN\left(x\right)=\Phi \circ x=\left[\sum_{i=1}^{d_{in}}w_{i,1}F_{\left\lfloor i∕d_{g}\right\rfloor }\left(x_{i}\right)\cdots \sum_{i=1}^{d_{in}}w_{i,d_{out}}F_{\left\lfloor i∕d_{g}\right\rfloor }\left(x_{i}\right)\right]$$

where i denotes the index of the input channel. With g groups, each group contains $d=d_{in}∕g$ channels, where $\left\lfloor i∕d_{g}\right\rfloor$ represents the group index. After transformation, the above equation can be rewritten as:

(6) $$GR-KAN\left(x\right)=linear\left(group\_rational(x)\right)$$

In this form, the parameters can be shared across input channels. Consequently, the rational function can be directly applied to the input vectors, that is, to each grouped edge.

Within the KAT module, layer normalization is applied to stabilize the input distribution of each layer, preserving the spatial structure of the point cloud features while maintaining consistent feature scaling during training. Compared to standard MLPs, KAT can effectively capture local spatial dependencies in point clouds, thereby enhancing feature representation and reducing computational cost.

## 4. Experiment and Analysis

### 4.1. Experimental Setup

#### 4.1.1. Experimental Environment

The experimental setup employed in this study consists of an Intel Core i7-14700KF processor (Intel, Santa Clara, CA, USA) with 32 GB of RAM, an NVIDIA GeForce RTX 4070 Ti SUPER graphics card (NVIDIA, Santa Clara, CA, USA), and CUDA 11.8-enabled GPU acceleration. The deep learning framework is based on Python 3.8 and utilizes PyTorch version 2.0.0+cu118. Detailed parameter configurations are presented in Table 1.

All experiments were conducted three times using different random seeds (1643723704, 1919680557, 1995020035), and the mean and standard deviation of the results were computed. The Adam optimizer was employed with an initial learning rate of 0.001 and a weight decay coefficient of 0.01. A linear warm-up strategy was applied during the first 1000 iterations, followed by a multi-stage decay of the learning rate by a factor of 0.1 at the 30th epoch. Training continued for 36 epochs with a batch size of 2. Data augmentation was performed using the GlobalRotScaleTrans and RandomFlip3D strategies to improve the model’s generalization capability in complex traffic scenarios.

#### 4.1.2. Evaluation Metrics and Datasets

To evaluate the effectiveness of the CMPNet model, experiments were conducted on the SemanticKITTI [44] and nuScenes [45] datasets. The SemanticKITTI dataset is a widely recognized large-scale benchmark for point cloud semantic segmentation, specifically designed for scene understanding in autonomous driving. It comprises point cloud sequences collected from 22 real-world road scenes, which are further subdivided into 43,552 detailed scenes, encompassing a total of over 4.5 billion 3D points. Following the official dataset partitioning guidelines, sequences 00–07 and 09–10 are designated for training, sequence 08 serves as the validation set, and sequences 11–21 constitute the test set. The nuScenes dataset includes a test set containing 8130 samples, a validation set comprising 6019 samples, and an additional independent test set of 6008 samples.

To comprehensively assess the performance of the proposed model in point cloud semantic segmentation tasks, this study employs mean Intersection over Union (mIoU), Accuracy (acc), and Accuracy per Class (acc_cls) as the primary evaluation metrics. The integration of these metrics enables a thorough quantification of the model’s overall performance in point cloud segmentation, while also providing a robust foundation for an in-depth analysis of the network’s accuracy and generalization capabilities across diverse scenarios.

1. The mean Intersection over Union (mIoU) denotes the average Intersection over Union (IoU) computed for each class within the dataset and is widely employed to evaluate the extent of overlap between the predicted results and the corresponding ground truth. It is calculated by averaging the IoU values across all classes in the entire dataset. The specific formula for this computation is presented as follows:
  (7) $$IoU_{i}=\frac{{TP}_{i}}{{TP}_{i}+{FP}_{i}+{FN}_{i}}$$
  (8) $$mIoU=\frac{\sum_{i=1}^{C}{IoU}_{i}}{C}$$
  where C is the number of point cloud semantic categories, TP denotes the number of correctly predicted as belonging to the current category, FP indicates the number of incorrectly predicted as current category point clouds, and FN denotes the number of incorrectly predicted as belonging to other categories.

2. Accuracy (ACC) represents the proportion of correctly predicted samples relative to the total number of samples and is commonly utilized to evaluate the overall classification performance of the model. The calculation formula is provided as follows:

(9) $$acc=\frac{TP+TN}{TP+FP+FN+TN}$$

3. ACC_cls denotes the mean accuracy computed across all categories and is employed to evaluate the model’s prediction performance for each individual category. By analyzing the accuracy of each category separately, it is possible to more comprehensively assess the model’s predictive capability for different types of samples.

#### 4.1.3. Loss Function

The loss function employed in this study comprises two components: voxel loss and point loss. The overall loss function can be formally expressed as follows:

(10) $$L=L_{voxel}+L_{point},$$

For the voxel loss component $L_{voxel}$, this study utilizes a combination of weighted cross-entropy loss and Lovasz-Softmax loss to simultaneously optimize point-wise accuracy and IoU scores. For the point loss component $L_{point}$, only the weighted cross-entropy loss is employed to supervise model training.

### 4.2. Comparative Experiment

#### 4.2.1. Results on SemanticKITTI

To establish the baseline model for this study, we conducted experiments on all mainstream algorithms supported by the MMDetection3D framework that are suitable for outdoor point cloud semantic segmentation, including MinkUNetW16, MinkUNetW20, MinkUNetW32, Cylinder3D, SPVCNNW16, SPVCNNW20, SPVCNNW32, RangeNet++ and SalsaNext. All experiments were performed using the default configurations provided by the MMDetection3D framework and executed on the SemanticKITTI dataset within a standardized experimental environment.

The experimental results demonstrate that the Cylinder3D model achieves superior segmentation accuracy under identical experimental conditions, significantly outperforming the majority of comparison models with a mean Intersection over Union (mIoU) of 62.17%. Although its mIoU (62.17%) is marginally lower than that of MinkUNetW32 (62.30%), improving MinkUNet’s performance requires adjusting its network width, which incurs higher computational costs. Moreover, while the overall accuracy of Cylinder3D is slightly lower than some comparison models, this metric is sensitive to category imbalance. In contrast, mIoU offers a more comprehensive assessment of segmentation capability and is therefore more informative for evaluating performance in point cloud segmentation tasks. Consequently, Cylinder3D is selected as the baseline model for this study.

The results, summarized in Table 2, indicate that CMPNet outperforms several state-of-the-art models. Specifically, the proposed CMPNet model achieves the highest mean Intersection over Union (mIoU) of 64.98%, representing an improvement of 2.81% over the previously optimal Cylinder3D model, which relies on cylindrical partitioning of the point cloud. This result demonstrates that CMPNet possesses a stronger capability to capture semantic boundaries of diverse objects in complex scenes. In the acc_cls metric, which evaluates category discrimination ability, CMPNet also attains superior performance, outperforming Cylinder3D and other comparison models. Although CMPNet exhibits slightly lower overall accuracy (acc) than MinkUNet and SPVCNN, it still surpasses the Cylinder3D model in this metric. The substantial lead of CMPNet in mIoU and acc_cls, particularly mIoU as a comprehensive metric reflecting balanced segmentation across categories, provides strong evidence that CMPNet excels in segmenting small objects, edge regions, and non-dominant categories, thereby achieving the most comprehensive and balanced segmentation performance. As detailed in Table 2, CMPNet demonstrates significant improvements over the original Cylinder3D model across multiple categories. CMPNet achieves remarkable advances in the segmentation of small and challenging objects, such as bicycles, motorcycles, and bicyclists, while simultaneously attaining optimal performance in critical road infrastructure categories, including road, sidewalk, and traffic signs.

**Figure 5:**
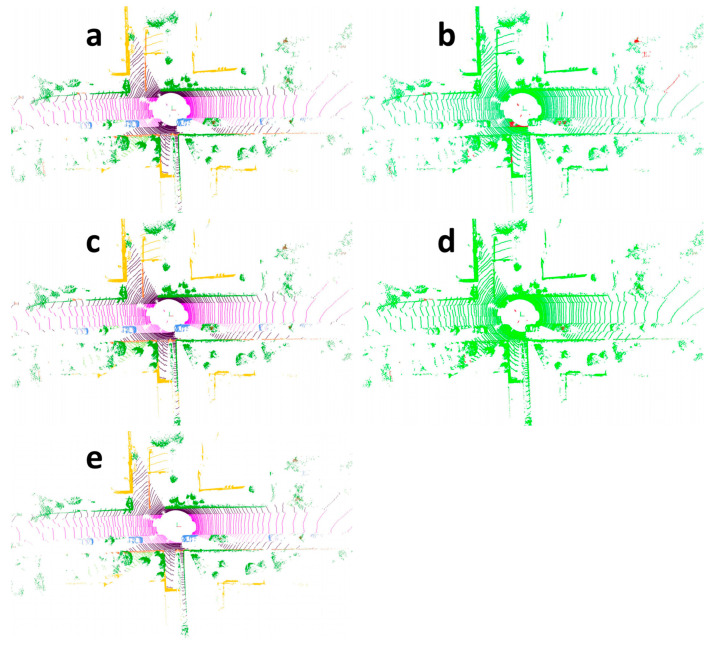
Point cloud semantic segmentation result visualization. (**a**) Visualization of Cylinder3D point cloud segmentation. (**b**) Two-color visualization of Cylinder3D point cloud segmentation. (**c**) Visualization of CMPNet point cloud segmentation. (**d**) Two-color visualization of CMPNet point cloud segmentation. (**e**) The ground truth segmentation.

To more intuitively demonstrate the segmentation performance, Figure 5 visualizes the segmentation results on the SemanticKITTI dataset. Specifically, Figure 5a,c illustrate the segmentation outputs of the Cylinder3D model and the CMPNet model, respectively, whereas Figure 5e depicts the ground truth segmentation. It is evident that the CMPNet model achieves higher semantic segmentation accuracy across most regions. In contrast, Cylinder3D tends to misclassify parts of parking areas as sidewalks and certain portions of buildings as fences. The CMPNet model, however, performs particularly well in accurately recognizing categories such as roads, trees, and roadside buildings. It not only effectively delineates various regions but also correctly distinguishes between different object classes. To provide a more direct comparison of model performance, Figure 5b,d present point cloud segmentation results using a two-color visualization scheme: red regions indicate discrepancies between the predicted results and the ground truth, whereas green regions represent areas of agreement. These visualization results clearly demonstrate that the proposed CMPNet model (Figure 5d) exhibits substantial advantages in point cloud semantic segmentation relative to the baseline model (Figure 5b). Although our method achieves strong overall performance, it may produce inaccurate segmentation results for distant objects. For instance, as shown on the rightmost side of Figure 5d, the CMPNet model fails to clearly distinguish the ground from the surrounding trees.

**Figure 6:**
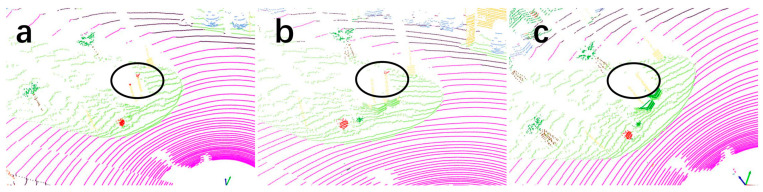
Visualization of CMPNet and Cylinder3D for Traffic Sign Recognition. (**a**) The ground truth segmentation. (**b**) Visualization of CMPNet point cloud segmentation. (**c**) Visualization of Cylinder3D point cloud segmentation.

Figure 6 presents a comparison of semantic segmentation results for traffic signs in the same scene using Cylinder3D and CMPNet, alongside the corresponding ground-truth labels. Specifically, Figure 6a depicts the ground-truth segmentation, while Figure 6b,c show the segmentation results produced by CMPNet and Cylinder3D, respectively. As illustrated, CMPNet demonstrates superior accuracy in recognizing traffic signs, exhibiting finer segmentation and enhanced robustness compared to Cylinder3D. This visual result is consistent with the quantitative experimental results on the SemanticKITTI dataset, where CMPNet achieves a mean Intersection over Union (mIoU) of 50.95% for the traffic sign category, demonstrating its enhanced capability in segmenting small and challenging objects.

#### 4.2.2. Results on nuScenes

To further validate the effectiveness of CMPNet, this study conducts generalization experiments on nuScenes [45], a publicly available large-scale dataset widely used for autonomous driving research. The results are summarized in Table 3. Despite the inherent complexity of the nuScenes dataset, which poses substantial challenges for point cloud segmentation, CMPNet continues to demonstrate outstanding performance. Specifically, the experimental results indicate that CMPNet achieves a mean Intersection over Union (mIoU) of 73.9%, corresponding to a 3.1% improvement over the Cylinder3D model. These findings not only further corroborate CMPNet’s performance superiority in point cloud semantic segmentation for outdoor traffic scenarios but also highlight its robust stability across different datasets.

To ensure the statistical reliability of our experimental results and mitigate the influence of randomness in training, each model was trained three times using different random seeds (1643723704, 1919680557, 1995020035). Table 2 and Table 3 indicate that CMPNet achieved an average mIoU of 64.98 ± 0.5 on the SemanticKITTI dataset and 73.9 ± 0.5 on the nuScenes dataset, demonstrating consistent model performance. Furthermore, a paired t-test comparing CMPNet with the baseline model Cylinder3D yielded *p* < 0.05, confirming that the observed performance improvement is unlikely to result from random variation.

### 4.3. Ablation Experiment

#### 4.3.1. Ablation Studies Between Different Modules

To evaluate the contribution of each module to the overall performance of CMPNet, this study employs the Cylinder3D model as the baseline and conducts a series of ablation experiments on the SemanticKITTI dataset. The results of these experiments are presented in Table 4. When the MS3DAM module is incorporated, the model’s mean Intersection over Union (mIoU) increases substantially to 64.04%, indicating that MS3DAM possesses a significant advantage in capturing both global features and complex spatial relationships within the point cloud. In contrast, adding only the PointMamba module raises the mIoU to 63.44%. Although this improvement is smaller than that achieved by MS3DAM, the PointMamba module’s strength lies in its state-space model with linear computational complexity, which enables efficient handling of large-scale point cloud data. Additionally, the use of the KAT module alone results in a 1.58% increase in mIoU. When modules are combined, further gains are observed. As both the PointMamba and MS3DAM modules are integrated simultaneously, the mIoU further rises to 64.31%, demonstrating that the synergistic combination of these two modules enhances the model’s feature extraction capability. When the KAT module is combined individually with either the PointMamba or MS3DAM modules, the mIoU improves further, with both combinations surpassing 64%. Ultimately, the complete CMPNet model, integrating all three modules, achieves an mIoU of 64.98%, corresponding to a 2.81% increase over the baseline. These findings fully substantiate both the effectiveness of each module and the synergistic benefits of their combined application in addressing complex point cloud semantic segmentation tasks.

#### 4.3.2. Multi-Scale Dilation Branch Ablation Experiment

To verify the effectiveness of the multi-scale dilation branches within the MS3DAM module, ablation experiments were conducted on its three individual dilation branches, and the results are presented in Table 5. Utilizing the CMPNet model as the baseline, the dilation rate of each branch was reduced independently, and experiments are conducted with various combinations of these rates. The experimental results indicate that the existing configuration of dilation rates in the MS3DAM module yields the optimal model performance, while modifying the dilation rate of any branch results in a significant decline in performance. These findings conclusively demonstrate that the synergistic interaction of the multi-scale branches plays a crucial role in enhancing the overall performance of the model.

#### 4.3.3. Attention Mechanism Ablation Experiment

To further analyze the contribution of the attention mechanisms in the MS3DAM module, we performed ablation experiments based on CMPNet. The model performance was evaluated using only channel attention, using only spatial attention, and disabling both attention mechanisms (No Attention), respectively, while keeping the multiscale expansion branch constant. The experimental results are shown in Table 6. The experimental results show that disabling both channel attention and spatial attention (No Attention) leads to a significant performance degradation (61.65%), while the performance improves to 64.24% and 64.15% when using only channel attention or only spatial attention, respectively. The best performance is achieved only when both channel and spatial attention are used (64.98%). This fully demonstrates that both channel attention and spatial attention contribute positively to the model performance, and their combination can achieve the optimal feature representation effect.

#### 4.3.4. Training Stability and Robustness Analysis

**Figure 7:**
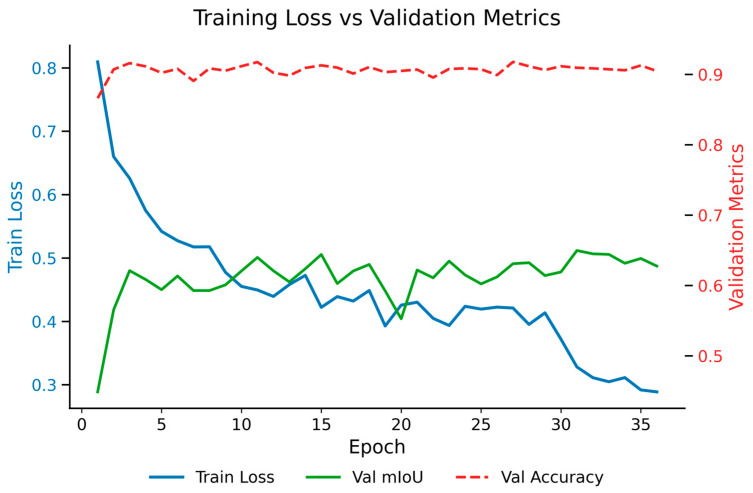
Training loss and validation performance metrics of CMPNet.

To further verify the robustness and generalization ability of the proposed CMP-Net, the training and validation processes were analyzed, as shown in Figure 7. The training loss exhibits a continuous downward trend, while both validation mIoU and accuracy remain stable and high throughout the training process. This indicates that CMPNet achieves steady convergence without significant overfitting. The close alignment between the training and validation curves demonstrates the model’s robust learning behavior and stable optimization across epochs. These observations further confirm that CMPNet maintains consistent performance under complex outdoor scenes, effectively mitigating overfitting while preserving high segmentation accuracy.

### 4.4. Computational Efficiency Analysis

To assess CMPNet’s computational efficiency, we evaluate its complexity in terms of the number of model parameters and compare it with the widely adopted baseline model, Cylinder3D. As presented in Table 7, CMPNet contains substantially fewer parameters than Cylinder3D, demonstrating that our design effectively reduces model redundancy while preserving robust representational capacity, and meanwhile attains the highest mIoU performance.

## 5. Discussion

In this paper, to address the limitations of the Cylinder3D algorithm, including erroneous segmentation, limited recognition capability, and low computational efficiency during the segmentation process, we propose an innovative semantic segmentation method for point clouds, CMPNet, which achieves high segmentation accuracy on the SemanticKITTI dataset through integrating the PointMamba, MS3DAM, and KAT modules. During the study, we observed that combining multi-scale dilated convolution with channel and spatial attention effectively expands the receptive field and captures spatial information at multiple scales. Consequently, the MS3DAM module was designed to enhance feature representation and strengthen the model’s ability to extract global features. The incorporation of the PointMamba module improves both segmentation accuracy and computational efficiency in point cloud semantic segmentation tasks. Moreover, experimental results indicate that introducing the KAT module at the head of the encoder allows CMPNet to exhibit superior perceptual capability and robustness when processing point clouds. Therefore, in comparison with the Cylinder3D algorithm, CMPNet demonstrates enhanced performance in recognizing complex scene structures and achieves overall improved robustness. The experimental results demonstrate that CMPNet outperforms advanced models such as Cylinder3D, MinkUNet, SPVCNN, and RangeNet++ in overall segmentation performance, achieving more comprehensive and balanced results.

Although CMPNet demonstrates strong segmentation accuracy on the SemanticKITTI dataset, several limitations were observed during the experiments. In particular, CMPNet tends to underperform in scenarios involving distant objects, where the MS3DAM module struggles to capture sufficient multi-scale contextual information in low-density regions, leading to fragmented or missing segmentation results. These failure cases indicate that further improvements in feature aggregation and attention calibration are needed to better handle irregular point cloud distributions. Moreover, while CMPNet improves segmentation accuracy, it also increases model complexity and computational overhead. Future research will continue to explore model lightweighting strategies to reduce computational costs while maintaining segmentation performance, thereby enhancing the practical applicability of CMPNet in traffic scene understanding. It should be noted that the current experiments may involve certain dataset-specific biases. To further assess and improve CMPNet’s generalization capability, future work will evaluate the model on a wider variety of datasets.

## 6. Conclusions

This study proposes CMPNet, an improved point cloud semantic segmentation framework built upon the Cylinder3D architecture, systematically addressing its limitations in multi-scale feature extraction, long-range dependency modeling, and robustness under sparse point cloud conditions. CMPNet integrates a newly designed Multi-Scale 3D Attention Module (MS3DAM) to enhance local and global feature representation, a KAT encoder to replace the traditional voxel feature encoder for improved geometric preservation, and the PointMamba module to efficiently model long sequences with linear computational complexity. Experimental results demonstrate that CMPNet attains robust segmentation performance on the publicly available SemanticKITTI dataset. Ablation studies further confirm the contribution of each individual module in enhancing model performance. The results indicate that CMPNet exhibits superior capability in recognizing complex scene structures and achieves greater overall robustness compared to the Cylinder3D algorithm. Future research will continue to focus on enhancing computational efficiency and improving the model’s ability to process sparse data, thereby enabling it to handle a broader range of real-world application scenarios.

## Figures and Tables

**Table 1:** Experimental Hyperparameter Settings.

Hyperparameters	Value
Grid_size	480 × 360 × 32
Optimizer	Adam
Batch size	2
Epoch	36
Momentum	0.1
Learning rate	0.001

**Table 2:** Comparison of recognition accuracy.

Method	mIoU	acc	acc_cls	Car	Bicycle	Motor	Truck	Bus	Person	Bicyclist	Road	Parking	Sidewalk	Building	Fence	Vegetation	Trunk	Terrain	Pole	Sign
MinkUNetW16	58.17	91.90	65.50	95.86	3.31	49.98	68.67	53.29	59.57	61.91	93.07	45.91	80.32	90.76	62.28	88.41	67.65	75.58	62.69	44.69
MinkUNetW20	60.58	**92.26**	67.20	96.46	11.91	49.05	78.70	62.58	62.12	74.66	93.14	46.79	80.28	90.53	61.35	**89.21**	**68.85**	**77.52**	62.88	43.64
MinkUNetW32	62.30	91.98	68.70	96.83	19.27	65.10	83.71	**67.66**	66.00	68.11	93.46	47.42	80.82	90.78	62.55	88.09	68.62	74.39	63.11	45.78
Cylinder3D	62.17	89.76	68.41	96.89	40.62	62.56	85.12	59.48	75.03	86.85	92.80	36.28	77.02	89.29	53.06	84.77	60.72	63.54	**65.82**	49.77
SPVCNNW16	59.63	92.02	67.14	96.25	8.35	49.64	71.13	58.53	62.63	75.96	92.74	44.79	79.78	90.51	61.65	89.00	66.87	76.69	62.31	45.85
SPVCNNW20	61.58	92.03	68.58	96.42	12.48	57.53	80.64	62.71	63.73	81.79	92.94	47.56	80.06	90.64	61.64	88.76	67.96	75.96	62.21	46.04
SPVCNNW32	61.88	91.94	68.99	96.26	21.85	60.45	78.06	59.26	66.22	77.04	92.83	**49.89**	79.84	**90.94**	**63.06**	88.50	68.26	74.93	62.26	45.19
RangeNet++	51.2	81.8	58.1	89.4	26.5	48.4	33.4	41.1	54.8	69.4	92.9	37.0	32.5	83.4	51.0	45.3	54.0	68.1	49.8	34.0
SalsaNext	59.4	90.0	66.3	90.5	44.6	49.6	**86.3**	35.4	74.0	81.4	93.4	40.6	36.3	84.6	53.0	48.0	64.3	64.2	54.4	39.8
CMPNet	**64.98** ** ± 0.5**	90.96	**71.30**	**96.98**	**50.59**	**72.10**	82.51	65.10	**76.74**	**90.92**	**94.47**	43.73	**81.20**	88.64	51.16	85.96	67.61	68.79	65.81	**50.95**

**Table 3:** Results of different methods on the nuScenes dataset.

Method	mIoU	Barrier	Bicycle	Bus	Car	Vehicle	Motorcycle	Pedestrian	Traffic cone	Trailer	Truck	Surface	Other flat	Sidewalk	Terrain	Manmade	Vegetation
RangeNet++	65.5	66.0	21.3	77.2	80.9	30.2	66.8	69.6	52.1	54.2	72.3	94.1	66.6	63.5	70.1	83.1	79.8
PolarNet	71.0	74.7	28.2	85.3	90.9	35.1	77.5	71.3	58.8	57.4	76.1	96.5	71.1	74.7	74.0	87.3	85.7
SalsaNext	72.2	74.8	34.1	85.9	88.4	42.2	72.4	72.2	63.1	61.3	76.5	96.0	70.8	71.2	71.5	86.7	84.4
Cylinder3D	70.8	71.1	28.0	84.2	86.7	39.4	57.8	72.9	59.7	53.1	79.9	96.3	70.7	73.6	74.5	87.2	86.3
CMPNet	**73.** **9** ** ± 0.5**	74.7	38.1	89.3	87.8	42.7	75.7	77.4	64.0	60.9	79.4	96.5	70.3	74.9	75.6	87.9	87.3

**Table 4:** Ablation experiment results.

PointMamba	MS3DAM	Kat	mIoU (%)
			62.17
√			63.44
	√		64.04
		√	63.75
√	√		64.31
√		√	64.27
	√	√	64.16
√	√	√	64.98

**Table 5:** Multi-scale dilation branch ablation experiment.

CMPNet	Branch2 Dilation Rate (3→1)	Branch3 Dilation Rate (12→3)	Branch 4 Dilation Rate (39→12)	mIoU (%)
√	√			62.42
√		√		64.26
√			√	64.89
√	√	√		63.69
√	√		√	64.81
√		√	√	64.54
√	√	√	√	64.98

**Table 6:** Attention mechanism ablation experiment.

CMPNet	No Attention	Channel Attention Only	Spatial Attention Only	mIoU (%)
√	√			61.65
√		√		64.24
√			√	64.15
√				64.98

**Table 7:** Comparison of model efficiency and segmentation performance on the SemanticKITTI.

Method	Params(MB)	mIoU
MinkUNet	14.24	62.30
SPVCNN	14.24	61.88
Cylinder3D	56.78	62.17
CMPNet	**14.12**	**64.98**

## Data Availability

The raw data supporting the conclusions of this article will be made available by the authors on request.
